# Effects of Interval Time of the Epley Maneuver on Immediate Reduction of Positional Nystagmus: A Randomized, Controlled, Non-blinded Clinical Trial

**DOI:** 10.3389/fneur.2019.00304

**Published:** 2019-04-04

**Authors:** Takao Imai, Tomoko Okumura, Takashi Sato, Noriaki Takeda, Yumi Ohta, Suzuyo Okazaki, Hidenori Inohara

**Affiliations:** ^1^Department of Otorhinolaryngology – Head and Neck Surgery, Osaka University Graduate School of Medicine, Suita, Japan; ^2^Department of Otorhinolaryngology – Head and Neck Surgery, Tokushima University Graduate School of Medicine, Tokushima, Japan; ^3^Department of Otolaryngology, Osaka City General Hospital, Osaka, Japan

**Keywords:** canalolithiasis, Epley maneuver, Dix-Hallpike test, BPPV fatigue, positional nystagmus

## Abstract

**Objective:** The Epley maneuver (EM) has an immediate effect: rapid reduction of positional nystagmus. Benign paroxysmal positional vertigo (BPPV) causes BPPV fatigue, which constitutes fatigability of positional nystagmus and vertigo with repeated performance of the Dix-Hallpike test; notably, BPPV fatigability becomes ineffective over time. We hypothesized that the immediate effect of the EM is caused by BPPV fatigue. Therefore, we suspected that performance of the EM with intervals between head positions would worsen the immediate reduction of positional nystagmus in patients with BPPV, because BPPV fatigability would become ineffective during performance of this therapy.

**Methods:** Forty patients with newly diagnosed BPPV were randomly assigned to the following two groups; one group performed the EM without intervals between positions (group A), and the other group performed the EM with 3 min intervals between positions (group B). The primary outcome measure was the ratio of maximum slow-phase eye velocity (MSPEV) of positional nystagmus soon after the EM, compared with that measured before the EM. Secondary outcome included whether a 30 min interval after the EM enabled recovery of MSPEV of positional nystagmus to the original value. This study followed the CONSORT 2010 reporting standards.

**Results:** In both groups A and B, the immediate effect of the EM could be observed, because MSPEV during the second Dix-Hallpike test was significantly smaller than MSPEV during the first Dix-Hallpike test (*p* < 0.0001 in group A, *p* < 0.0001 in group B). The primary outcome measure was larger in group B than in group A (*p* = 0.0029). The immediate effect faded 30 min later (secondary outcome).

**Conclusions:** This study showed that the EM had an immediate effect both with and without interval time in each head position of the EM. Because setting interval time in each head position of the EM reduced the immediate effect of the EM, interval time during the EM adds less benefit. This finding can reduce the effort exerted by doctors, as well as the discomfort experienced by patients with pc-BPPV, during EM. However, this immediate effect may be caused by BPPV fatigue, and may fade rapidly.

**Classification of Evidence:** 1b

## Introduction

After performance of the Epley maneuver (EM) for treatment of benign paroxysmal positional vertigo (BPPV) (1), otoconia are in the utricle; thus, in 70% of patients with BPPV, positional nystagmus cannot be observed when the Dix-Hallpike test (2) is performed immediately after the EM (3). The immediate effect of the EM is rapid reduction of positional nystagmus. A characteristic finding of BPPV is known as BPPV fatigue, which constitutes fatigability of positional nystagmus with repeated performance of the Dix-Hallpike test (4, 5). BPPV fatigue is generally considered to occur due to dispersal of otoconia within the affected canal, thus reducing cupular deflection with positioning; conversely, the therapeutic effect of EM is related to the removal of otoconia from the affected canal, which is presumably returned to the utricle/main vestibule (6–8). Notably, both the EM and BPPV fatigue can reduce positional nystagmus. Hence, we hypothesized that the immediate effect of the EM is caused by BPPV fatigue. In our previous study, we showed that the positional nystagmus reduced by BPPV fatigue recovered to its original intensity after 30 min (5). If our hypothesis were correct, we would expect the following results. First, positional nystagmus reduced after the EM would return to its original intensity after 30 min because of recovery from BPPV fatigue. Second, the intensity of residual positional nystagmus after performance of the EM over an extended period of time would be larger than that after performance of the EM over a short period of time, because positional nystagmus reduced by BPPV fatigue can partially recover during performance of the EM over an extended period of time. By including interval time between head positions of the EM, the effects of the EM may be extended in length. As a result, residual positional nystagmus is expected to increase when intervals are included in the EM. This study aimed to show that interval time is not necessary in each position of the EM.

## Materials and Methods

The protocol of this study is available at the following website: http://www.med.osaka-u.ac.jp/pub/ent/speciality/files/cr_deafness161215.pdf. This experiment was performed at the Department of Otorhinolaryngology – Head and Neck, Osaka University Hospital, in accordance with the Declaration of Helsinki. Before the experiment, written informed consent was obtained from all participants. Approval for the study was obtained from the ethics committee of our hospital (No. 16165-2), and the study was registered in the University Hospital Medical Information Network (UMIN000025291). This study was a single-center, non-blinded, randomized controlled clinical trial. It was designed on the basis of the 2010 Consolidated Standards of Reporting Trials.

**Figure 1:**
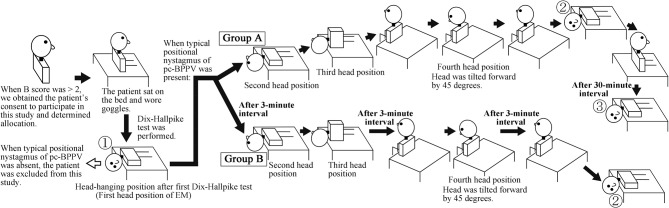
Schema of this study (when the right side is affected). ①: First Dix-Hallpike test, ②: Second Dix-Hallpike test, ③: Third Dix-Hallpike test. At the head positions ①, ②, and ③, positional nystagmus was recorded. At head position ①, positional nystagmus was recorded when the diagnosis of posterior canal type of benign paroxysmal positional vertigo at head position ①; thus, patients' heads were continuously turned 90° to the unaffected ear at the second position of the Epley maneuver (EM), in order to transition immediately from the Dix-Hallpike test to the EM.

The target patients were patients with posterior canal type of BPPV (pc-BPPV) (2). In this study, we measured the maximum slow-phase eye velocity (MSPEV) of positional nystagmus induced by the Dix-Hallpike test (performed when we diagnosed patients with pc-BPPV) and then quickly performed the EM after diagnosing patients with pc-BPPV (9); therefore, prior to diagnosis of pc-BPPV, we obtained each patient's consent to participate in this study and then performed patient allocation (Figure 1). Thus, we chose patients who were strongly suspected of BPPV solely by medical interview and then diagnosed them with pc-BPPV after obtaining consent.

We previously developed a new scoring system for the diagnosis of BPPV via patient interview, which we described as the “BPPV score (B score)” (10). The details of this scoring system are provided in Appendix 1. The B score can be used to identify patients who are likely to exhibit BPPV, prior to performing Dix-Hallpike test for diagnosis of pc-BPPV. Therefore, in this study, the inclusion criteria were as follows: outpatients (from 20 to 95 years old) at our hospital whose complaint was dizziness and/or vertigo (D/V) and whose B score was greater than two. The exclusion criteria were as follows: patients who did not consent to participate in this study, patients with medical history or possibility of cervical or lumber disease, patients with severe heart disease, patients with possible pregnancy, and pregnant women. After patients provided consent for participation, three authors (TI, YO, and SO) enrolled patients in the study; another author (TS) performed patient allocation. The three doctors were certified to have expert knowledge and medical technique to treat patients with complaint of D/V; this certification was provided by the Japan Society for Equilibrium Research. Patients were randomly allocated to either group A or B by block randomization. The block size of randomization was decided by the author (TS), with a size of four; only TS knew the size. In Group A, the EM was performed (9, 11) without interval time between head positions. In Group B, the EM was performed with 3 min interval times between head positions. The details are shown in Figure 1. In group A, during EM, when positional nystagmus could be observed at each head position of EM, the patient's head was moved to the next head position soon after eliminating positional nystagmus. When the positional nystagmus was not eliminated within 1 min at head positions other than the first head position of the EM (① in Figure 1), the patient's head was moved to the next head position at 1 min after the nystagmus appeared. When positional nystagmus was not eliminated within 1 min at the first head position of the EM (① in Figure 1), the patient was excluded from this study because the patient's pathophysiology was cupulolithiasis, not canalolithiasis (2, 12). When positional nystagmus could not be observed at head positions other than the first head position of the EM, the patient's head was moved to the next head position after approximately 30 s. In group B, in addition to the EM method used for group A, 3 min interval times were provided at the first, third, and fourth head positions. Three minute interval times were not provided at the second head position because the EM for group B was based on the EM approach in the book, “Vertigo,” which has been read by doctors who treat patients with complaints of D/V (11). In the EM approach described in that book, a 3 min interval is not provided at the second position. After data collection for 20 patients with pc-BPPV was completed in one group, all patients who met the inclusion criteria were allocated to the other group. Only TS knew when data collection of 20 patients was completed in either group.

After determining their group allocation, the patients wore goggles with an infrared charge-coupled device camera (RealEyes; Micromedical Technologies, Chatham, IL, USA). If a patient said that they felt stronger D/V with the left ear downwards than with the right ear downwards in supine position, the patient initially performed the left Dix-Hallpike test. If not, the patient initially performed the right Dix-Hallpike test. When a patient did not show typical positional nystagmus (as observed in patients with pc-BPPV) during the initial Dix-Hallpike test, with a strong torsional component in the direction of the lower ear (2, 13), the patient performed the Dix-Hallpike test on the other side. Eye movement during the Dix-Hallpike test was recorded on a Windows computer using USB-connected video capture, GV-USB2 (I-O DATA, Ishikawa, Japan); patients were instructed to keep their eyes open and to continue looking forward in darkness during the test. When both left and right Dix-Hallpike tests did not reveal typical positional nystagmus (as observed in patients with pc-BPPV), such patients were excluded from this study; they then underwent regular medical examination. When the typical positional nystagmus was observed (① in Figure 1), patients' heads were continuously moved to the second head position of the EM; that method was performed in accordance with the group allocation for each patient. Soon after completing the EM, the affected side Dix-Hallpike test was again performed (② in Figure 1) and the eye movement during the test was recorded. Group A patients performed a third affected side Dix-Hallpike test, 30 min after the second Dix-Hallpike test (③ in Figure 1). The positional nystagmus during the third Dix-Hallpike test was recorded. We asked patients to stand with minimal movement during the 30 min between the second and third Dix-Hallpike tests. After data collection, if patients desired, we administered another EM to the patients. By using video editing software, Adobe Premiere Pro CS4 (Adobe Systems Incorporated, San Jose, CA, USA), one of authors (TI) captured only the eye movement induced by Dix-Hallpike tests, from the eye movement-recorded movies in all patients. The captured movies were provided in random order to another author (TO) who was blinded to the patients' data. TO analyzed the eye movement three-dimensionally and determined the MSPEV of positional nystagmus.

### Three-Dimensional Analysis of Eye Movements

The 30-Hz movies of positional nystagmus induced by Dix-Hallpike test were converted to 720 × 480-pixel JPEG images and analyzed with an algorithm developed in our laboratory (14). Details of the method of three-dimensional analysis of eye movements are given as Appendix 2. To calculated the axis angle of eye velocity around the X-, Y-, and Z-axes (15, 16). She then extracted the slow-phase eye velocity (SPEV) of the nystagmus using a fuzzy set-based approach (17, 18) and determined the MSPEV of positional nystagmus (5).

### Primary and Secondary Outcomes

The primary outcome measure was the ratio of MSPEV of positional nystagmus in the second Dix-Hallpike test (② in Figure 1) compared with that of the first Dix-Hallpike test (① in Figure 1). This ratio is an indicator of the immediate effect of the EM: when the ratio is small, the immediate effect is good; when the ratio is large, the immediate effect is poor. The secondary outcome measure was MSPEV of positional nystagmus induced by the third Dix-Hallpike test, which was performed 30 min after the second Dix-Hallpike test in group A (③ in Figure 1).

### Method for Determining the Target Number of Patients

In our preliminary study, as written in the protocol of this study (http://www.med.osaka-u.ac.jp/pub/ent/speciality/files/cr_deafness161215.pdf.), in group A, the average ratio of MSPEV of positional nystagmus when second Dix-Hallpike test against that when first Dix-Hallpike test (primary outcome measure) was 0.3. In group B, the average ratio was 0.7. The standard deviation of the ratio was 0.45. These results were reported in the protocol of this study. The difference to be detected is set to be 0.4 (0.7–0.3), α error is set to be 0.05 (two-sided test), and statistical power, 1-β, is set to be 80%. From the following formula, *n* = 2^*^0.45^2^(1.960+0.842)^2^/0.4^2^, we decided the number of patients in one group was 20, and the total number of patients was 40.

### Statistical Analysis

Statistical analysis was performed using BellCurve for Excel (Social Survey Research Information Co., Ltd, Tokyo, Japan). In group A, differences in MSPEVs of positional nystagmus were compared for statistical significance by using the Friedman test, a non-parametric test for testing the differences among several related samples; the Wilcoxon signed-rank test was used as a *post hoc* test. In group B, differences in MSPEVs of positional nystagmus were compared for statistical significance by using the Wilcoxon signed-rank test. Differences in primary outcome measure between groups A and B were compared for statistical significance by using the *t*-test because the statistical analysis method of the primary outcome measure was decided before data collection by the protocol. Statistical significance was defined as a *p-*value < 0.05.

### Data Sharing Statement

Movies showing eye movement induced by Dix-Hallpike tests, captured from the eye movement-recorded movies of all patients, are stored at Department of Otorhinolaryngology – Head and Neck, Osaka University Graduate School of Medicine. Access to the movies is available upon approval by the ethics committee of Osaka University Hospital.

## Results

**Figure 2:**
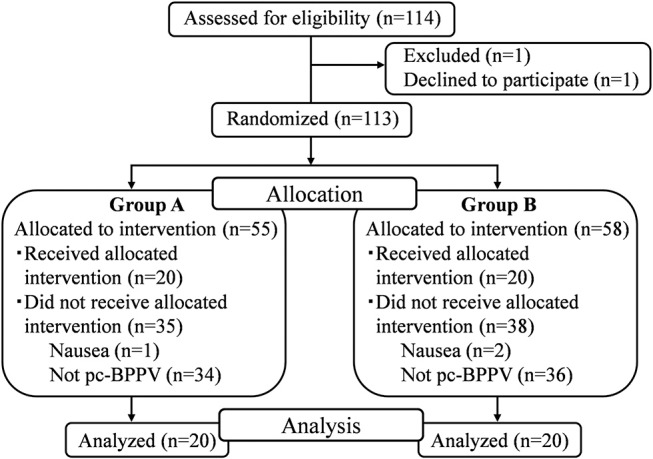
Patient flow through the study. The positional nystagmus of 40 patients with posterior canal type of benign paroxysmal positional vertigo was analyzed.

**Figure 3:**
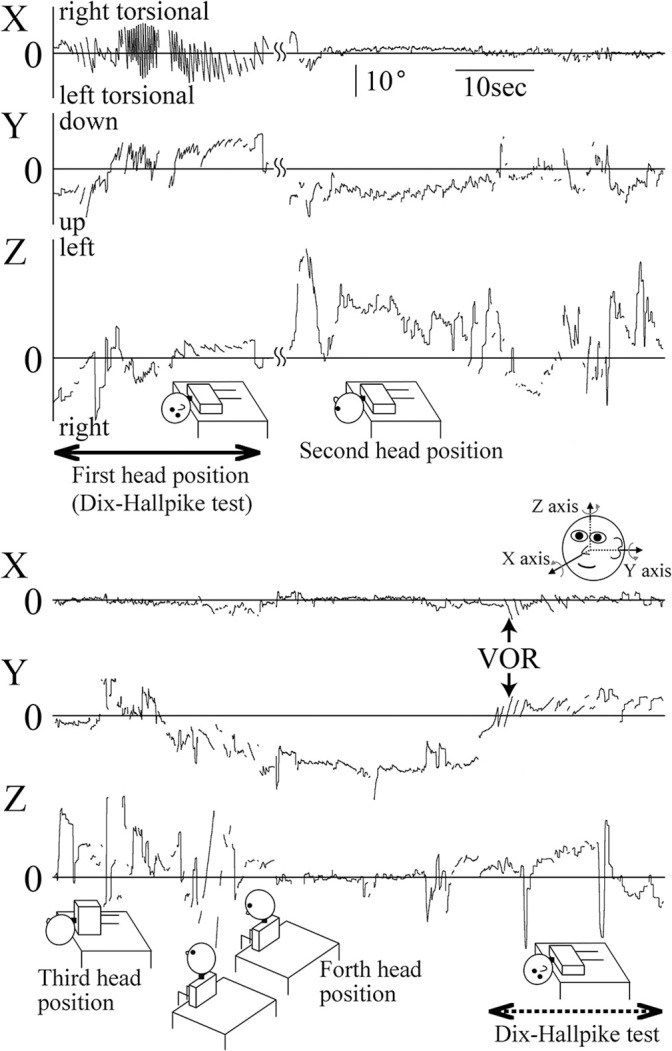
Three-dimensional axis angles of eye position of a representative patient who was allocated to group A during the Epley maneuver (EM) and the following Dix-Hallpike test. Highest column: X component (torsional component), middle column: Y component (vertical component), lowest column: Z component (horizontal) component. Because this figure contains data that were recorded during the head movement of EM, positional nystagmus and nystagmus induced by vestibulo-ocular reflex during the head movement could be observed. At the part indicated by a solid double headed arrow, the head was at that position where we observed positional nystagmus induced by the first right Dix-Hallpike test. From this head position, next, the head was moved to the second head position of the EM. Therefore, this head position is also the first head position of the EM. At this part, very strong positional nystagmus could be seen, especially in X and Y components. The positional nystagmus was right torsional and upward attenuated nystagmus. This positional nystagmus was typical of nystagmus seen in patients with right side-affected posterior canal type of benign paroxysmal positional vertigo (13). At the second head position, very weak downward nystagmus could be seen (Y component). At the third head position, no clear nystagmus could be seen. At the fourth head position, very weak downward nystagmus could be seen (Y component). At the following second right Dix-Hallpike test, the part was shown by dotted double head arrows, very weak right torsional and upward positional nystagmus could be seen. Note that a positive Y component indicates downward rotation, while a negative Y component indicates upward rotation; a positive Z component indicates leftward rotation, while a negative Z component indicates rightward rotation.

**Figure 4:**
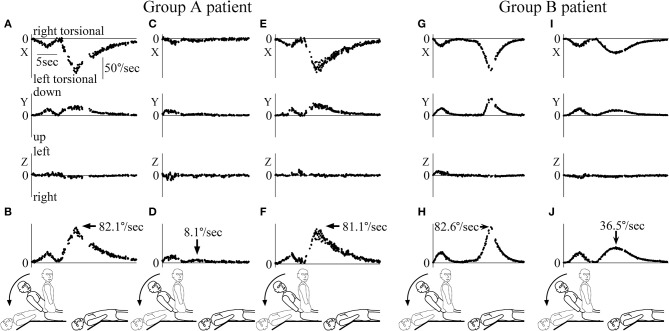
Three-dimensional axis angles of slow phase eye velocity (SPEV) during and after the head movement of the Dix-Hallpike test. The scales of all graph are identical to that shown in **(A)**. **(A)** Three-dimensional axis angle of SPEV of the patient whose data are shown in Figure 3 during the first Dix-Hallpike test. Highest column: X component (torsional component), middle column: Y component (vertical component), lowest column: Z component (horizontal component). These data were calculated from the axis angles of eye position data during the first Dix-Hallpike test, at the part shown by a solid double head arrow in Figure 3. **(B)** SPEV around the eye rotational axis calculated from the data shown in **(A)**. **(C)** Three-dimensional axis angle of SPEV of the patient whose data are shown in Figure 3 during the second Dix-Hallpike test. These data were calculated from the axis angles of eye position data during the second Dix-Hallpike test, at the part shown by a dotted double head arrow in Figure 3. The clear peak of positional nystagmus seen in **(A)** could not be seen either in X or Y component. **(D)** SPEV around the eye rotational axis calculated from the data shown in **(C)**. **(E)** Three-dimensional axis angle of SPEV of the patient whose data are shown in Figure 3, during the third Dix-Hallpike test. **(F)** SPEV around the eye rotational axis calculated from the data shown in **(E)**. The value of the maximum SPEV (MSPEV) was almost same with the value of MSPEV during the first Dix-Hallpike test shown in **(B)**. **(G)** Three-dimensional axis angle of SPEV of a patient who was allocated to group B during the first Dix-Hallpike test. **(H)** SPEV around the eye rotational axis calculated from the data shown in **(G)**. **(I)** Three-dimensional axis angle of SPEV of the patient whose data are shown in **(G,H)** during the second Dix-Hallpike test. **(J)** SPEV around the eye rotational axis calculated from the data shown in **(I)**. The value of the MSPEV became approximately half of the value of MSPEV shown in **(H)**.

The first patient was included in the study on December 22, 2016 and the final patient was included in the study on April 5, 2018. The inclusion of patients to the study was completed on April 5, 2018 because we completed acquisition of data from 40 patients with pc-BPPV. A flowchart describing patient allocation and analysis is shown in Figure 2. A total of 114 patients met the inclusion criteria, and one patient declined to participate in this study. Of the remaining 113 patients, 55 were randomized to group A and 58 were randomized to group B. Of these 113 patients, 70 withdrew because their diagnosis was not pc-BPPV and three withdrew because of nausea. Because the nausea in all three patients faded within 1 h, this study could be completed without harm. Finally, the positional nystagmus of 40 patients with pc-BPPV was analyzed. Differences in sex, age, affected side, and MSPEV of positional nystagmus before performance of the EM among patients were not statistically significant. Hence, the data were comparable, as shown in the Table 1. The quality of images of positional nystagmus from all 40 patients was adequate for analysis. Figure 3 shows the three-dimensional axis angle of eye position of positional nystagmus when the EM was performed for right side-affected pc-BPPV, and during a second Dix-Hallpike test following the EM, for one patient (78 years of age, female, right ear affected) who was allocated to group A. During the first Dix-Hallpike test (first head movement of the EM) at the part indicated by a solid double-headed arrow (↔), the direction of the positional nystagmus was right torsional (X component), upward (Y component) and leftward (Z component). During a second Dix-Hallpike test at the part indicated by a dotted double-head arrow, the nystagmus induced by vestibulo-ocular reflex could be clearly observed, but the positional nystagmus was very weak. In Figures 4A,B, the axis angle of SPEV of the patient during the first Dix-Hallpike (at the part indicated by a solid double-headed arrow in Figure 3) is shown. Two peaks could be observed: the first peak was made by vestibulo-ocular reflex during the head movement of the Dix-Hallpike test; the second peak was the point at which the intensity of positional nystagmus was strongest. The value of the axis angle of SPEV (SPEV of positional nystagmus around the axis of eye rotation) is shown in Figure 4B. The value is calculated from the data shown in Figure 4A:

$$\begin{array}{l} \sqrt{{\left(the\;value\;of\;X\;component\right)}^{2}+{\left(the\;value\;of\;Y\;component\right)}^{2}+{\left(the\;value\;of\;Z\;component\right)}^{2}} \end{array}$$

**Table 1:** Baseline data of patients in the two groups.

	**Group A (*N* = 20)**	**Group B (*N* = 20)**	**Between group comparison**
Age (years) (median)	70.5 (34–84)	75 (39–86)	*p* = 0.3845 (t test)
Sex (female / male)	12/8	10/10	*p* = 0.5250 (chi-square test)
Affected side (right / left)	10/10	9/11	*p* = 0.7515 (chi-square test)
MSPEV when first Dix-Hallpike test (°/s) (median)	47.9 (8.1–98.9)	49.0 (18.8–103.9)	*p* = 0.7508 (*t*-test)

*Data are shown as median value (minimum value – maximum value)*.

The second peak (indicated by an arrow) shows the MSPEV of positional nystagmus (82.1°/s). The axis angle of SPEV of the patient during the second Dix-Hallpike test, performed soon after EM (at the part indicated by dotted double-headed arrow in Figure 3) is shown in Figures 4C,D. The intensity of positional nystagmus was weak in all three components. The value of the axis angle of SPEV is shown in Figure 4D. The MSPEV of positional nystagmus was 8.1°/s at the part indicated by the arrow in Figure 4D. The ratio of MSPEV during the second Dix-Hallpike test against MSPEV during the first Dix-Hallpike test was 0.099 (8.1/82.1). The ratio is the primary outcome measure of the patient. The axis angle of SPEV of positional nystagmus of the patient during the third Dix-Hallpike test, performed 30 min after the second Dix-Hallpike test, is shown in Figures 4E,F. The value of axis angle of SPEV is shown in Figure 4F. The MSPEV was 81.1°/s. The MSPEV is the second outcome measure of the patient. In Figures 4G–J, in another patient (79 years of age, female, right ear affected) who was allocated to group B, the axis angles of SPEV of positional nystagmus during the first Dix-Hallpike test (Figures 4G,H) and second Dix-Hallpike test (Figures 4I,J) are shown. The value of the primary outcome measure of the patient was 0.442 (36.5/81.1).

**Figure 5:**
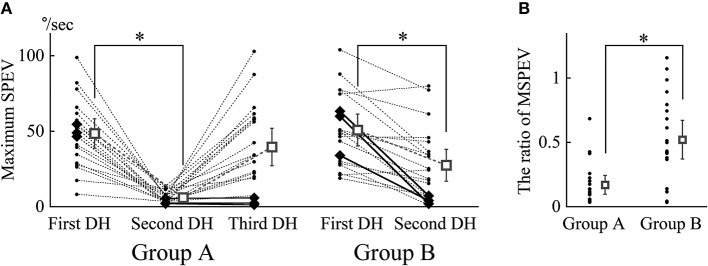
The maximum slow phase eye velocity (MSPEV) of positional nystagmus induced by Dix-Hallpike tests and the ratio of the MSPEV during the second Dix-Hallpike test against the MSPEV during the first Dix-Hallpike test (*p < 0.05). **(A)** MSPEV of positional nystagmus during all Dix-Hallpike tests in all patients. DH: Dix-Hallpike test. In group A, there was a significant difference among MSPEV during the first, second, and third Dix-Hallpike tests by Friedman's test (*p* < 0.0001). In both groups A and B, the MSPEV during the second Dix-Hallpike test was significantly lower than that during the first Dix-Hallpike test. However, in group A, the MSPEV during the third Dix-Hallpike test was not significantly different from that during the first Dix-Hallpike test. The MSPEVs for an individual patient were connected by dotted lines. In group A, large rhombuses show the data of three patients, whose MSPEV during the third Dix-Hallpike test was <15% of the MSPEV during the first Dix-Hallpike test. In group B, large rhombuses show the data of three patients, whose MSPEV during the second Dix-Hallpike test was <15% of the MSPEV during the first Dix-Hallpike test. The square shows the averaged MSPEV; error bars show the 95% confidence interval. **(B)** The ratio of the MSPEV during the second Dix-Hallpike test against the MSPEV during the first Dix-Hallpike test (primary outcome measure). The ratio in group B was statistically higher than the ratio in group A.

All MSPEVs of positional nystagmus of all patients are shown in Figure 5A. In both groups A and B, the immediate effect of the EM could be observed, because in both groups MSPEV during the second Dix-Hallpike test was significantly smaller than MSPEV during the first Dix-Hallpike test (*p* < 0.0001 in group A, *p* < 0.0001 in group B). However, the immediate effect of the EM was eliminated 30 min after the EM in group A because there was no statistical difference between MSPEV during the first Dix-Hallpike test and MSPEV during the third Dix-Hallpike test in group A (*p* = 0.1913).

Using these MSPEV, we calculated the ratio of MSPEV during the second Dix-Hallpike test against MSPEV during the first Dix-Hallpike test (the ratio is the primary outcome measure) in groups A and B; this is shown in Figure 5B. The immediate effect of the EM in group B was worse than that in group A because the ratio in group B was statistically higher than the ratio in group A (*p* = 0.0029).

## Discussion

This study showed that the EM had an immediate effect both with and without interval time in each head position of the EM because the intensity of positional nystagmus (MSPEV) became significantly smaller after the EM (Figure 5A). However, setting interval time in each head position of the EM reduced the immediate effect of the EM because the ratio of MSPEV of positional nystagmus after the EM, compared with that before the EM with interval time (group B), was higher than that without interval time (group A) (Figure 5B). The evidence for the good immediate effect of the EM, that the 3 min interval time in each head position of the EM is not needed, is shown by this study. However, the good immediate effect of the EM without the interval time (group A) faded within 30 min after performance of the EM (Figure 5A).

The results of this study can be explained by using the hypothesis that the immediate effect of the EM is caused by BPPV fatigue, rather than by recession of otoconia back to the utricle (1). As shown in our previous study, BPPV fatigue has the effect of reducing MSPEV of positional nystagmus; this effect recedes after 30 min (5). Thus, positional nystagmus that is reduced by BPPV fatigue gradually recovers to the original intensity within 30 min. According to the above hypothesis, when the performance time of the EM was 10–15 min, as in group B, the effect of BPPV fatigue faded gradually during the EM. Therefore, the positional nystagmus that was reduced by BPPV fatigue recovered to some extent after the EM in group B. However, in group A, the effect of BPPV fatigue remained after the EM because the procedure time of the EM was short for this group. As the result, the ratio of MSPEV of positional nystagmus during the second Dix-Hallpike test, compared with that during the first Dix-Hallpike test, was larger in group B than in group A. This explains the result of the primary outcome measure. According to the above hypothesis, it is reasonable that the MSPEV of positional nystagmus 30 min after the EM recovered to the value of MSPEV of positional nystagmus before the EM in group A because the effect of BPPV fatigue faded after 30 min. This explains the result of the second outcome measure. The findings that the MSPEV of positional nystagmus during the second Dix-Hallpike test in group A was smallest (average MSPEV was 6.0°/s), that during the second Dix-Hallpike test in group B was middle (average MSPEV was 27.5°/s), and that during the third Dix-Hallpike test in group A was largest (average MSPEV was 39.5°/s) (Figure 5A) can be explained by the idea that the effect of BPPV fatigue during the second Dix-Hallpike test in group A is strongest, that during the second Dix-Hallpike test in group B is of moderate intensity, and that during the third Dix-Hallpike test in group A is weakest.

We have attempted to explain the results of this study by using the theory of the EM (1). It is possible to explain the result of the second outcome measure as follows. The otoconia transferred back to the utricle during the EM enters the duct of the posterior canal again via the ampulla of the posterior canal over 30 min. However, it is very difficult to explain the result of the primary outcome measure, for the following reason: according to the theory of the EM, the success of the EM depends on the second head position (Figure 1) (7). At the second head position, when otoconia moves in the correct direction into the common crus, the EM is successful; conversely, when otoconia moves backward into the cupula, the EM is unsuccessful (19). The results of the primary outcome measure indicate that more otoconia moved backward into the cupula in group B than in group A. However, because the interval time of the second position in group B was identical to that in group A, i.e., no interval time (Figure 1), it is unlikely that the difference of the movement of otoconia between in group A and B was caused at the second head position. Therefore, before the second head position (i.e., at first head position), the otoconia may not have moved suitably in group B; however, this is unlikely. At the first head position, otoconia only moves in the ampullofugal direction in the posterior canal by means of gravity; thus, a longer interval at the first head position should allow the otoconia to move more clearly in the ampullofugal direction in the posterior canal. For the above reasons, it is difficult to explain the result of the primary outcome measure by using the theory of the EM. In this study, the second Dix-Hallpike test was performed soon after EM, which may affect the results of this study. However, because in groups A and B, the second Dix-Hallpike test was performed under the same conditions (i.e., soon after EM), the findings clearly showed that the immediate effect of EM in group B was worse than that in group A.

Figure 5A shows that in three patients of group A, the ratio of MSPEV during the third Dix-Hallpike test against MSPEV, compared with that during the first Dix-Hallpike test, remained <15% (indicated by large rhombus). Moreover, Figure 5A shows that in three patients of group B, the ratio of MSPEV during the second Dix-Hallpike test, compared with that during the first Dix-Hallpike test, remained <15% (indicated by large rhombus). In these six patients, otoconia in the posterior canal may return to the utricle by the EM because the positional nystagmus remained weak, although BPPV fatigue should fade over time. From these results, it is possible that in most patients (85%, 34/40), the reduction of positional nystagmus was caused by BPPV fatigue, rather than by the therapeutic effect of the EM itself. However, it is possible that in these six patients, the effect of BPPV fatigue continued even an extended period of time; as shown in our previous study, two of 20 patients exhibited the effect of BPPV fatigue after 30 min (5).

In group B, our preliminary study had shown that the intensity of positional nystagmus induced by the third Dix-Hallpike test was nearly identical to that induced by the second Dix-Hallpike test. Thus, we did not perform the third Dix-Hallpike test for patients allocated to group B. Notably, there was no statistically significant difference between the MSPEV of positional nystagmus induced by the second Dix-Hallpike test in group B and that induced by the third Dix-Hallpike test in group A (Figure 5A, *p* = 0.1850, Mann-Whitney *U*-test).

The effect of the EM highlights some timing differences in executing the EM. Notably, this timing difference is often contingent on the clinical interaction between the attending physician (e.g., his/her experience in executing the EM) and the patient characteristics (e.g., age, size/weight, and level of tolerance to vertigo). However, in this study, we consider this effect to be negligible because the study was randomized and controlled, in order to reduce such bias. As shown in the Table 1, there were no differences in patient characteristics between groups A and B. In addition, the three physicians (TI, YO, and SO) had sufficient knowledge and skill with regard to the EM, as certified by the Japan Society for Equilibrium Research. In order to support the conclusion that the immediate effect of the EM is caused by BPPV fatigue, it is essential that the three physicians' knowledge and skill with regard to the EM are sufficient. Indeed, the intensity of positional nystagmus became smaller after the EM in almost all patients (Figure 5).

The positional nystagmus of pc-BPPV has gaze-dependent differential effects on the direction of induced eye movements (11); this is crucial, because it can easily change MSPEV. However, the present study did not need to consider the effect of gaze direction on MSPEV because we instructed patients to maintain a forward gaze (“straight ahead”) during the test. If patients did not follow this instruction, we did not need to consider the effect of gaze direction on MSPEV for the following reasons. Many studies validate Ewald's first law in humans: the axis of nystagmus should match the anatomic axis of the semicircular canal that generated the nystagmus (20). Thus, the eye rotates around the anatomic axis of the affected posterior canal, independently of gaze direction, when positional nystagmus is induced by the Dix-Hallpike test in patients with pc-BPPV. In this study, because we analyzed the axis angle (rotation vector) of SPEV, we analyzed the eye angular velocity around the axis of eye rotation (i.e., around the axis of the affected posterior canal) (Figures 4B,D,F,H,J) (13). Therefore, the MSPEVs in this study were not affected by gaze direction during positional nystagmus. In addition, if SPEV around the axis of the affected posterior canal was affected by gaze direction, we did not need to consider the effect of gaze direction because this study was randomized and controlled, in order to reduce such bias. Indeed, as shown in the Table 1 and in Figure 5, there was no difference in the MSPEVs when the first Dix-Hallpike test was conducted in groups A and B.

In this study, we used MSPEV as the index of the intensity of positional nystagmus because it was easy to extract MSPEV from SPEV of positional nystagmus. We did not use the duration time of positional nystagmus as the index of the intensity of positional nystagmus because we could not calculate the exact duration time for the following reasons. In order to calculate the duration time of positional nystagmus exactly, we must determine the start and end points of positional nystagmus. However, it is very difficult to determine both points. As shown in Figures 3, 4, before the start point of positional nystagmus, SPEV was induced by vestibulo-ocular reflex during the head movement of the Dix-Hallpike test; moreover, the end of the SPEV overlapped with the start of the SPEV of positional nystagmus. Thus, it is difficult to extract the start point of positional nystagmus from the overlapping portion. Furthermore, the theoretical value of SPEV at the end point is 0°/s. Therefore, when SPEV is 0°/s, this constitutes the end point. However, the value of SPEV of positional nystagmus is almost 0°/s around the end point, as shown in Figure 4; thus, it is difficult to clearly determine which value of SPEV is 0°/s.

A limitation of this study is that it was not double-blinded. However, this should not affect the result of this study, in that patients knew which their group allocation. This is because they could not control the intensity of positional nystagmus, despite knowing their group. Bias was not involved when analyzing MSPEV of positional nystagmus because one author (TO), who was blinded to patient information, analyzed the MSPEVs.

In this study, when analyzing the axis angles of eye movement, we used our own video-oculography (VOG) system. Therefore, when a similar study is done in another hospital and the eye movement is analyzed by the VOG system that is used in the hospital, the value of MSPEVs during the Dix-Hallpike test may differ from the values shown in this study. However, the results indicate that the ratio of MSPEV of positional nystagmus during the second Dix-Hallpike test, compared with that measured during the first Dix-Hallpike test, is larger in group B than in group A; there was no difference of MSPEV between the first and third Dix-Hallpike tests in group A.

## Conclusion

This study showed that, to achieve the immediate effect of the EM, the 3 min interval between head positions of the EM is unnecessary. This finding can reduce the effort exerted by doctors, as well as the discomfort experienced by patients with pc-BPPV, during the EM. However, because this immediate effect of the EM without interval time fades after 30 min, there is a possibility that the immediate effect for reduction of positional nystagmus is caused by BPPV fatigue, rather than by the established therapeutic effect of the EM itself. The conclusions are derived only from the results of immediate effect of EM. In the future, we will investigate whether similar conclusions can be made based on the results of extended effects of EM (i.e., whether the effect is maintained at 1 day or 1 week after the performance of EM).

## Contribution to the Field Statement

This study is a randomized, controlled, and non-blinded clinical trial that was designed on the basis of the 2010 Consolidated Standards of Reporting Trials. This study assessed the Epley maneuver, which constitutes treatment of posterior canal type of benign paroxysmal positional vertigo and consists of four sequential head positions; the study showed that the Epley maneuver had an immediate effect both with and without intervals between head positions of the maneuver. Because the use of an interval between head positions of the Epley maneuver reduced the immediate effect of the maneuver, the use of intervals during the Epley maneuver may not be beneficial. This finding can reduce the effort exerted by doctors, as well as the discomfort experienced by patients with posterior canal type of benign paroxysmal positional vertigo, during the Epley maneuver. The results of this study suggest that the immediate effect of the Epley maneuver is caused by benign paroxysmal positional vertigo fatigue, rather than by the established therapeutic effect of the Epley maneuver itself. This fatigue is a characteristic finding of benign paroxysmal positional vertigo, which constitutes fatigability of positional nystagmus with repeated performance of the positional nystagmus test.

## Author Contributions

TI substantially contributed to conception of the study and drafting of the article. TI, TS, YO, and SO substantially contributed to acquisition of data. TI and TO substantially contributed to analysis of data. TI and NT substantially contributed to interpretation of data. HI substantially contributed to study supervision.

### Conflict of Interest Statement

The authors declare that the research was conducted in the absence of any commercial or financial relationships that could be construed as a potential conflict of interest.
