# Supplementary material for: Effects of Interval Time of the Epley Maneuver on Immediate Reduction of Positional Nystagmus: A Randomized, Controlled, Non-blinded Clinical Trial
Source: Front Neurol. 2019 Apr 4;10:304. doi: 10.3389/fneur.2019.00304 (PMC6459130; doi:10.3389/fneur.2019.00304)
Supplement: Supplementary file 1 [file Data_Sheet_1.docx]

**Appendix 1**

***B score***

We developed a new scoring system for the diagnosis of benign paroxysmal positional vertigo (BPPV) via patient interview (10). It consists of the following four questions: (i) Is rotary vertigo a characteristic of your dizziness and/or vertigo (D/V)? (ii) Is your D/V triggered when you roll your head over in a supine position? (iii) Does your D/V disappear within 5 min? (iv) Have you previously experienced hearing loss in one ear, or have you experienced hearing loss, tinnitus, or ear fullness with this D/V? One point each is given to an answer of “yes” to questions (i) and (ii). Two points are given to an answer of “yes” to question (iii). One point is subtracted upon an answer of “yes” to question (iv). A total score (BPPV score (B score)) greater than two points is associated with positional nystagmus in more than 80% of patients; thus, patients with this score are diagnosed with BPPV. Therefore, before performing the Dix-Hallpike test, by using these four questions, we can identify patients who are strongly suspected to exhibit BPPV. Because the B score is designed to identify patients who are likely to exhibit BPPV, it identifies patients who exhibit posterior canal type of BPPV (pc-BPPV), as well as patients who exhibit other types of BPPV. For this study, we designed a scoring system for the diagnosis of pc-BPPV via patient interview. Our previous study attempted to develop this type of scoring system, but was unsuccessful for the following reasons (21). Patients with pc-BPPV experience vertigo when their heads are moved in the sagittal plane because this head movement stimulates the posterior canal. Patients with horizontal canal type of BPPV (hc-BPPV) experience vertigo when their heads are moved in the axial plane because this head movement stimulates the horizontal canal. Therefore, pc-BPPV can be distinguished from hc-BPPV by determining whether vertigo is induced by head movement in the sagittal or axial planes. However, this difference between pc-BPPV and hc-BPPV cannot be easily determined because many patients with pc-BPPV, as well as those with hc-BPPV, stated that head movements in both sagittal and axial planes induced vertigo (Higashi-Shingai K et al. Acta Otolaryngol (2011) 131:1264-9). Thus, we could not develop a scoring system for the diagnosis of pc-BPPV via patient interview. As shown in Fig. 2, the dropout rate of patients was high in the present study. This appears unavoidable because there is no existing method to identify only patients with pc-BPPV solely on the basis of interviews. In this study, the ratio of pc-BPPV in patients whose B score was greater than 2 was 38% (43/113), which seems low. However, this ratio is similar to that reported in our previous study (10). A potential explanation is as follows: the BPPV that can be identified by B score includes all types of BPPV in which positional nystagmus can be observed, such as pc-BPPV (canalolithiasis and cupulolithiasis), hc-BPPV (canalolithiasis and cupulolithiasis), and anterior canal type of BPPV (10). The ratio of pc-BPPV in all types of BPPV was 50% in our previous study (10). The ratio of identifying BPPV by using B score was 81% in the previous study; therefore, the ratio of pc-BPPV in patients whose B score was greater than 2 was 41% (0.81×0.5), which was similar to the ratio of 38% in the present study.

It may seem unusual that one of the questions focuses on head movement in the axial plane (“Is your D/V triggered when you roll your head over in a supine position?”), rather than head movement in the sagittal plane, despite our intention to solely identify patients with pc-BPPV. However, this question is appropriate because many non-BPPV patients experience vertigo solely upon head movement in the sagittal plane; as described above, patients with pc-BPPV experience vertigo upon both head movements in both sagittal and axial planes (10, Higashi-Shingai K et al. Acta Otolaryngol (2011) 131:1264-9). Therefore, by using this question (“Is your D/V triggered when you roll your head over in a supine position?”), we can identify patients with pc-BPPV and exclude non-BPPV patients; notably, we cannot avoid identifying patients with hc-BPPV. Conversely, if we use a different question (e.g., “Is your D/V triggered when you sit up and/or lie down on a bed, and/or when you look up/down?”), we can identify patients with pc-BPPV, but cannot avoid identifying either non-BPPV patients or patients with hc-BPPV.

In addition, other two questions (“Does your D/V disappear within 5 minutes?” and “Have you previously experienced hearing loss in one ear, or have you experienced hearing loss, tinnitus, or ear fullness with this D/V?”) may appear unusual for identifying patients with pc-BPPV because “5 minutes” is excessive and because hearing loss is not helpful in the diagnosis of BPPV. However, these two questions are also appropriate because, as described above, we cannot avoid identifying patients with hc-BPPV. Therefore, we must exclude non-BPPV patients. Many non-BPPV patients exhibit vertigo with a duration of more than 5 minutes, as well as hearing loss, tinnitus, or ear fullness (10); thus, these two questions can be used to avoid identifying non-BPPV patients.

**Appendix 2**

***Three-dimensional analysis of eye movements***

The head coordinates were reconstructed in three dimensions and defined as follows: the X-axis was parallel to the naso-occipital axis (positive forward), the Y-axis was parallel to the interaural axis (positive left), and the Z-axis was normal to the X-Y plane (positive upwards) (Appendix figure 1A). The 30-Hz movies of positional nystagmus induced during Dix-Hallpike test were converted to 720 × 480-pixel JPEG images and analyzed with an algorithm developed in our laboratory (14). The two-dimensional coordinates of the center of the pupil (yp zp) and an iris freckle (yi zi) in the image were determined (Appendix figure 1B) (5). The edge of the pupil was also detected and approximated using an ellipse. We then found the minor and major axes of the ellipse. The center of eye rotation (*o*) (yc zc) on the image plane was determined as the intersection of the extensions of the minor axes (Appendix figure 1C) (14, Imai T et al. Exp Brain Res (2017) 235:2575-90). After determining the center of eye rotation, we calculated the radius of rotation of the center of the pupil (*R*) using the following formula: $R\sqrt{1-\left( {the length of minor axis}/{the length of major axis} \right)^{2}}=d$

Here, *d* is the length between *o* and the center of the pupil ellipse, *p* (Fig. 6D). Next, we calculated the length of the radius of rotation of an iris freckle (*R’*). We reconstructed the three-dimensional coordinates of the center of pupil and an iris freckle in the head-fixed coordinate system as $\left( \begin{matrix} \sqrt{R^{2}-\left( yp-yc \right)^{2}-\left( zp-zc \right)^{2}} & yp-yc & zp-zc \end{matrix} \right)$ and $\left( \begin{matrix} \sqrt{{R'}^{2}-\left( yi-yc \right)^{2}-\left( zi-zc \right)^{2}} & yi-yc & zi-zc \end{matrix} \right)$. The relationship between the three-dimensional coordinates of the center of pupil and the iris freckle in the head, compared between test and reference positions, was used to calculate the rotation vector of the eye position, **r** (Haslwanter T. Vision Res (1995) 35:1727-39). The reference position was defined as the eye position when the participant’s gaze was “straight ahead” with their head in an upright position. A “straight ahead” gaze was defined as a target with a horizontal gaze in front of the eyes (Haslwanter T. Vision Res (1995) 35:1727-39). The X, Y, and Z components of the axis angle of the eye position primarily reflected the roll, pitch, and yaw components, respectively (Appendix figure 1A). The direction of rotation was described from the participants’ point of view. For the X-component, “right torsional” and “left torsional” indicated the superior pole of the eyeball rotated to the right and left ears, respectively. The accuracy of this method for analyzing eye rotation vectors has been described elsewhere (14, Imai T et al. Auris Nasus Larynx (2005) 32:3-9). We calculated the rotation vector of eye velocity around the X-, Y-, and Z-axes, **ω,** by the formula, **ω** = 2 × (d**r**/dt + **r** × d**r**/dt) / (1 + **r**^2^) (Haslwanter T. Vision Res (1995) 35:1727-39). We used the Euler angle parameter, given as 2 × tan^-1^(magnitude of rotation vector, **r**, **ω**), to represent eye position and velocity as an axis-angle representation (15, 16). We then extracted the slow-phase eye velocity (SPEV) of the nystagmus using a fuzzy set-based approach (17, 18) and determined the maximum SPEV of positional nystagmus (5).





**Supplementary Figure 1:** Three-dimensional analysis of eye movements. (A) Schema of the three-dimensional head coordinate frame. (B) Extraction of the two-dimensional coordinates of the center of the pupil and an iris freckle. Two-dimensional coordinates of an iris freckle are indicated by tip of black dotted triangle and two-dimensional coordinates of the center of the pupil are

indicated by a white cross. (C) Centre of eye rotation in a two-dimensional image plane. Ideally, multiple minor axes of a pupil ellipse intersect at a single point. The point is the center of eye rotation in a two-dimensional plane, o. (D) Calculation of the length of the radius of rotation of the center of the pupil. " is the eye rotation angle from the eye position during frontal vision. d is the length between o and the center of the pupil ellipse, labeled p in the image. R is the length of the radius of rotation of the center of the pupil.
